# Incidence and treatment of anastomotic leakage after esophagectomy in German acute care hospitals: a retrospective cohort study

**DOI:** 10.1097/JS9.0000000000002274

**Published:** 2025-01-29

**Authors:** Marie-Christin Weber, Nicolas Jorek, Philipp-Alexander Neumann, Jeannine Bachmann, Rebekka Dimpel, Marc Martignoni, Marcus Feith, Helmut Friess, Alexander Novotny, Maximilian Berlet, Daniel Reim

**Affiliations:** a Department of Surgery, Technical University of Munich, TUM School of Medicine and Health, TUM University Hospital, Klinikum rechts der Isar, Munich, Germany

**Keywords:** anastomotic healing, anastomotic leakage, esophagectomy, EVT, failure-to-rescue, gastrectomy, hospital volume, SEMS

## Abstract

**Background:**

Anastomotic leakage (AL) is a major concern following esophagectomy due to the associated morbidity and mortality. The impact of hospital volume on postoperative outcomes after esophagectomy has previously been reported. The aim of this study was to analyze the current trends in postoperative anastomotic leakage and associated failure-to-rescue after esophagectomy in relation to hospital volume in German acute care hospitals using real-world data from the German Diagnosis-Related Groups (G-DRG) database.

**Materials and methods:**

A retrospective secondary data analysis of the G-DRG database was performed for all in-hospital cases of patients undergoing esophagectomy from 2013 to 2021. AL and in-house mortality rates were assessed in relation to hospital case volume and endoscopic treatment modalities.

**Results:**

The study included 32 335 cases. The mean reported AL rate was 17.1% with a mean failure-to-rescue rate of 18.9%. AL rates did not differ between hospitals with an annual case-volume ≤ 25 procedures/year vs. >25 procedures/year (16.8% vs. 17.6%, OR 1.06, *P* = 0.07). However, in high-volume centers (> 25 procedures/year), in-hospital mortality for cases with AL (failure-to-rescue) was lower compared to medium-volume (10-25 cases/year) and low-volume (1-9 cases/year) centers (14.2% vs. 21.5% vs. 25.1%). The use of endoscopic vacuum therapy (EVT) increased over time, reaching 58.1% of AL cases in 2021 compared to 14.2% in 2013, while the use of self-expanding metal stents (SEMS) decreased from 37.0% in 2013 to 9.3% in 2021.

**Conclusions:**

AL rates after esophagectomy remain high. In-house mortality is significantly lower in high-volume hospitals highlighting the importance to consider improvements in centralization of procedures. Further efforts are needed to reduce AL rates and improve outcomes after esophagectomy.

## Introduction

Anastomotic leakage (AL) is one of the most common but also most detrimental surgical complications following esophagectomy for esophageal cancer. In addition to reducing the risk of postoperative respiratory and cardiac complications, the promotion of intact anastomotic healing and successful management of AL significantly determine postoperative outcomes^[1,2]^.

Reported AL rates in larger cohort studies and meta-analyses range from 6.3% to 19%^[3–8]^. AL is most commonly treated endoscopically with self-expanding metal stents (SEMS) or endoscopic vacuum therapy (EVT). EVT is increasingly being used, but recent meta-analyses show inconclusive results on whether its use is associated with reduced postoperative mortality^[9,10]^. Hence, prospective data from randomized controlled trials are still lacking.

Since the early 2000s, an inverse relationship between center-volume and postoperative mortality rates for esophagectomies has been apparent, with lower mortality rates in high-volume centers compared to low-volume centers^[11–13]^. More recent publications have repetitively shown similar results^[14–16]^. Using German population-based hospital reimbursement data, Nimptsch *et al* demonstrated that reduced postoperative mortality in high-volume centers is not related to an overall reduction in postoperative complications, but rather to the overall management of cases with postoperative complications[17]. As such, the Federal Joint Committee (Gemeinsamer Bundesausschuss, GBA), the highest decision-making joint self-government institution of German physicians, hospitals, and insurance funds, increased the minimum center volumes for complex esophageal surgeries/esophagectomies from >10 to >25 cases per year in 2023. However, centralization of complex surgical procedures is not always easy to implement, especially with regard to patient interests (easier access to local hospitals, etc.) and missing infrastructure of high-volume centers to manage increasing case volumes. In consideration of the current change in surgical techniques in esophageal resections with a shift from open surgery to laparoscopic and robotic-assisted procedures, there is a knowledge gap regarding the development of AL rates, associated mortality/failure-to-rescue, and their correlation with hospital volume. In particular, nationwide real-world data that not only cover cases operated on in large centers and university hospitals are currently not available for European countries and Germany specifically. Closing this knowledge gap may be of great importance in order to evaluate the effects of previous attempts to centralize esophageal surgery in Germany and to draw possible consequences for the further enforcement of centralization of complex esophageal surgeries.

To gain more detailed information on this complex relationship between hospital volume, AL, and postoperative in-house mortality/failure-to-rescue, we performed a nationwide population-based study on AL after esophagectomy based on the German hospital reimbursement database (German Diagnosis-Related Groups (G-DRG) database). The focus of this study was also on current trends in endoscopic management of AL after esophagectomy and the assessment of the impact of SEMS and EVT.

## Methods

### Data access

Commonly accepted methods of secondary data analysis to approach the epidemiologic analysis of postoperative complications using data from the G-DRG database have been described in detail previously^[18–21]^. To summarize, the German Diagnosis-Related Groups (G-DRG) statistics provide a complete overview of hospital reimbursement data for all inpatient cases of every German acute care hospital. Data can be retrieved by remote-controlled data access through the Federal Statistical Office (DESTATIS). The G-DRG database contains non-longitudinal data on individual treatment cases from all acute care hospitals in Germany. The database contains case information including ICD (International Classification of Diseases)- and OPS (Operation and Procedure Classification System)-codes, data on the mode of discharge including in-house mortality, length of hospital stay, hospital reimbursement volume, and other hospital-related data per individual case.

The source code for the G-DRG database query was generated using the SAS programming language (SAS Base version 9.4), in accordance with the specifications as required by DESTATIS. Data were obtained for each year queried via remote-controlled data processing and delivered in aggregated form[22]. Study design, data analysis, and reporting were performed according to the recommendations of the Good Practice of Secondary Data Analysis (GPS), the German Reporting Standard for Secondary Data Analyses (STROSA) guidelines, and the STROCSS guidelines^[23–25]^ No ethics committee statement is required for the secondary data analysis used in this study. To ensure data protection, DESTATIS obscures case numbers ≤ 2, making them inaccessible to the authors.

### Patient criteria

All inpatient cases undergoing complex esophageal surgery with reconstruction (esophagectomy and esophagogastrectomy) between 2013 and 2021 in German acute care hospitals defined by the OPS-codes 5-424, 5-426, 5-427.0, 5-427.1, 5-438.0, and 5-438.1 (according to the definition by the Federal Joint Committee, GBA) were included for secondary data analysis. A detailed description of the OPS codes is provided in the Supplemental Online Material (Table S1, http://links.lww.com/JS9/D811).

Robotic-assisted procedures were defined by the additional OPS code 5-987.

Primary outcomes were AL (K91.83) and in-house mortality rates in relation to the hospital volume and the use of endoscopic procedures. Hospital volume was defined by the number of cases of complex esophageal surgery with reconstruction per year, as defined above. Three categories were defined: ≤ 9 cases/year (low-volume centers), 10–25 cases/year (medium-volume centers), and >25 cases/year (high-volume centers) according to the old (>10 cases/year) and the new (>25 cases/year) minimum volume standard defined by the German Federal Joint Committee. The use of endoscopic vacuum therapy (EVT) and endoscopic stenting (self-expandable metal stents, SEMS) as a secondary outcome was defined by the OPS-codes 5-916.a and 5-429.j.

Secondary outcomes were the case mix reimbursement volume (mean hospital reimbursement sum per case) and the length of hospital stay. The Charlson Comorbidity Index (CCI) was used for the assessment of general comorbidity[26]. Additionally, secondary diagnoses, diabetes (E11.-), obesity (E66.-), cachexia (R64), hypertension (I10.-), chronic kidney disease (N18.-), chronic heart disease (I25.-), COPD/Emphysema (J43.-; J44.-), and asthma (J45) were assessed individually for their relation to AL rates (Table S1, http://links.lww.com/JS9/D811).

### Statistical analysis

Data visualization and statistical analysis were performed using GraphPad Prism (Version 10.1.0, GraphPad Software, CA, USA). Data were analyzed for each year, as only yearly statistics can be retrieved from the G-DRG database. AL and in-hospital mortality rates are presented as relative frequencies for each subgroup of patient cases per year as depicted in the graphs. Statistical tests were applied as described in the table and figure legends. A *P*-value <0.05 was considered as statistically significant.

## Results

### General anastomotic leakage rates

**Figure 1.**
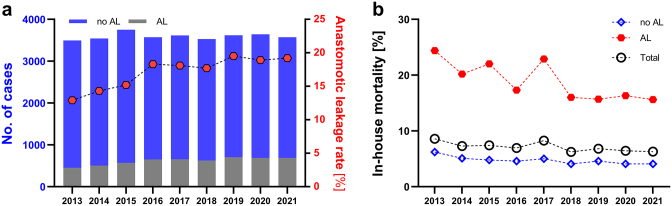
Case numbers, anastomotic leakage, and in-house mortality rates after esophagectomy. (a) Number of cases without (blue) and with anastomotic leakage (AL) per year (left y-axis) and mean AL rates per year (red, right y-axis). (b) In-house mortality rates per year for cases without AL (blue), with AL (red), and the total number of cases per year (black).

A total number of 32 335 cases with esophagectomy were identified by the data query and included in the study. Case numbers for esophagectomy were stable over the study period from 2013 to 2021 with a mean of 3593 cases per year (3496 in 2013 and 3572 in 2021) (Fig. 1a, Table 1). The mean reported AL rate was 17.1% ± 2.4% (Table 1). From 2013 to 2016, reported AL rates were increasing as the ICD code K91.83 for AL was only introduced in the G-DRG database in 2013. From 2016 to 2021, reported AL rates per year ranged from 18.3% to 19.5% with a mean AL rate of 18.6% (Fig. 1a, Table 1). In-house mortality rates were more than three-fold higher in cases with AL than in cases without AL with a mean in-house mortality rate of 4.7% in cases without AL and 18.9% in cases with AL (Fig. 1b). The mean in-house mortality rate for all cases analyzed in this study was 7.1%. The overall in-house mortality rates decreased from 8.6% in 2013 to 6.3% in 2021.

**Table 1:** Overview of anastomotic leakage (AL) rates per year regarding center volume

		Center volume		
Year	Total number of surgeries	≤9 cases/year	10–25 cases/year	>25 cases/year
2013–2021
*n* (total)	32 335	6265	13 094	12 976
*n* (AL)	5540	1057	2198	2285
AL rate	17.1%	16.9%	16.8%	17.6%
2013
*n* (total)	3496	955	1448	1093
*n* (AL)	451	115	189	147
AL rate	12.9%	12.0%	13.1%	13.4%
2014
*n* (total)	3543	923	1301	1319
*n* (AL)	506	127	192	187
AL rate	14.3%	13.8%	14.8%	14.2%
2015
*n* (total)	3752	832	1581	1339
*n* (AL)	569	139	229	201
AL rate	15.2%	16.7%	14.5%	15.0%
2016
*n* (total)	3573	695	1524	1354
*n* (AL)	653	122	290	241
AL rate	18.3%	17.6%	19.0%	17.8%
2017
*n* (total)	3614	662	1489	1463
*n* (AL)	654	127	270	257
AL rate	18.1%	19.2%	18.1%	17.6%
2018
*n* (total)	3527	578	1423	1526
*n* (AL)	626	111	248	267
AL rate	17.7%	19.2%	17.4%	17.5%
2019
*n* (total)	3617	578	1469	1570
*n* (AL)	705	113	265	327
AL rate	19.5%	19.6%	18.0%	20.8%
2020
*n* (total)	3641	512	1461	1668
*n* (AL)	689	102	261	326
AL rate	18.9%	19.9%	17.9%	17.9%
2021
*n* (total)	3572	530	1398	1644
*n* (AL)	687	101	254	332
AL rate	19.2%	19.1%	18.2%	20.2%

We analyzed relevant secondary diagnoses in relation to AL and in-house mortality rates. Diabetes (OR 1.17, *P* = 0.0008), obesity (OR 1.34, *P* < 0.0001), hypertension (OR 1.16, *P* < 0.0001), chronic kidney disease (OR 1.35, *P* < 0.0001), and chronic heart disease (OR 1.21, *P* = 0.0005) as secondary diagnoses showed statistically significant association with AL. Meanwhile, diabetes (OR 1.38, *P* < 0.0001), cachexia (OR 1.47, *P* < 0.001), chronic kidney disease (OR 2.72, *P* < 0.0001), chronic heart disease (OR 1.94, *P* < 0.0001), and COPD/emphysema (OR 1.79, *P* < 0.0001) as secondary diagnoses showed a statistically significant association with in-house mortality (Table S3, http://links.lww.com/JS9/D811).

Unfortunately, the use of laparoscopic surgical techniques during esophagectomy is not represented in the G-DRG system and is thus not available for analysis in this study. However, the use of robotic assistance systems during surgery is represented by the OPS-code 5-987.0. The number of esophagectomies performed with a robotic assistance system was 3 (0.1%) in 2013 and 741 (20.7%) in 2021 (Fig. S1a, http://links.lww.com/JS9/D811). AL rates for esophagectomies performed with a robotic assistance system were available from 2014. However, reported AL rates were substantially higher in robotic procedures than in those without robotic assistance from 2014 to 2016 (29.6–32.0% vs. 14.2–18%) (Fig. S1a, http://links.lww.com/JS9/D811, Table S3, http://links.lww.com/JS9/D811). AL rates for robotic esophagectomy decreased in 2015 but were still higher than for non-robotic esophagectomy until the end of the study period in 2021 (22.9% vs. 18.3%) (Fig. S1b, http://links.lww.com/JS9/D811).

### Anastomotic leakage and mortality rates in relation to hospital case volume

**Figure 2.**
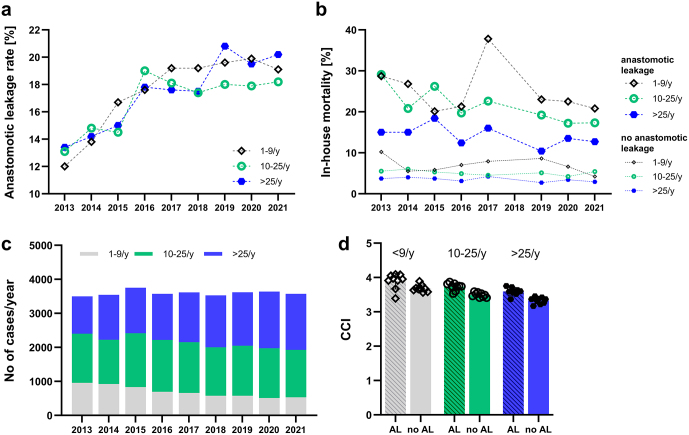
Anastomotic leakage and in-house mortality in relation to hospital case volume. (a) Anastomotic leakage (AL) rates per year for low volume centers (≤9 cases/year, black rhombus), medium volume centers (10–25 cases/year, green circle), and high-volume centers (> 25 cases/year, blue hexagon). (b) In-house mortality rates for low-, medium-, and high-volume centers and for cases with AL (large symbols) and without AL (small symbols). Data for 2018 not available. (c) Total number of cases per year treated in low-, medium-, and high-volume centers. (d) Charlson Comorbidity Index (CCI). Bars show the mean CCI for low-, medium-, and high-volume centers, and cases with and without anastomotic leakage. Dots are the mean CCI for each year 2013–2021.

The mean AL rates were similar in all three categories of hospital case volume with a mean AL rate of 16.9% in low-volume centers (≤9 cases/year), 16.8% in medium-volume centers (10–25 cases/year), and 17.6% in high-volume centers (>25 cases/year) (Fig. 2a, Table 1). However, when looking at the related in-house mortality rates, mortality was considerably lower in high-volume centers for both cases with and without AL (Fig. 2b). The mean in-house mortality rates for cases with AL were 25.1% in low-volume, 21.5% in medium-volume, and 14.2% in high-volume centers. Even in cases without AL, in-house mortality was lower in high-volume centers with the mean in-house mortality rates for cases without AL of 7.0% in low-volume, 5.1% in medium-volume, and 3.5% in high-volume centers. When comparing AL and in-house mortality rates between hospitals with an annual case volume ≤25 cases/year and >25 cases/year, the AL rate was not statistically significantly different (16.8% vs. 17.6%, OR 1.06, *P* = 0.07), whereas mortality rates were significantly lower in hospitals >25 cases/year (8.5% vs. 5.3%, OR 0.60, *P* < 0.0001) (Table 2). Even though a minimal center volume of 10 cases per year was introduced in Germany by the GBA in 2006, a high percentage of cases were still operated on in low-volume centers (26.3% in 2013, 14.8% in 2021, Fig. 2c). The CCI as a measure for general comorbidity was higher in low-volume centers than in high-volume centers with a mean CCI of 3.8 ± 0.22 in low, 3.7 ± 0.11 in medium, and 3.6 ± 0.11 in high-volume centers for cases with AL (Fig. 2d).

**Table 2:** Risk of anastomotic leakage and in-house mortality in relation to center volume

	Center volume	Anastomotic leakage					In-house mortality (all cases, with + without anastomotic leakage)				
Year	(cases/ year)	YES [*n*, (%)]	NO [*n*]	OR	95% CI	*P**^a^*	YES [*n*] [*n*, (%)]	NO [*n*]	OR	95% CI	*P**^a^*
**2013-2021**	**>25**	2285 (17.6)	10 691	1.06	0.99–1.12	0.07	603 (5.3)	10 847	0.60	0.54–0.66	**<0.0001**
	**≤25**	3255 (16.8)	16 104				1484 (8.5)	15 874			
2013	**>25**	147 (13.4)	946	1.07	0.86–1.33	0.55	57 (5.2)	1036	0.49	0.36–0.66	**<0.0001**
	**≤25**	304 (12.7)	2099				243 (10.1)	2160			
2014	**>25**	187 (14.2)	1132	0.99	0.81–1.20	0.93	73 (5.5)	1246	0.65	0.48–0.86	**0.003**
	**≤25**	319 (14.3)	1905				185 (8.3)	2039			
2015	**>25**	201 (15.0)	1138	0.98	0.81–1.19	0.88	79 (5.9)	1260	0.70	0.53–0.92	**0.01**
	**≤25**	368 (15.3)	2045				199 (8.2)	2214			
2016	**>25**	241 (17.8)	1113	0.95	0.79–1.14	0.6	65 (4.8)	1289	0.56	0.41–0.76	**0.0001**
	**≤25**	412 (18.6)	1807				183 (8.2)	2036			
2017	**>25**	257 (17.6)	1206	0.94	0.79–1.12	0.52	92 (6.3)	1371	0.63	0.49–0.82	**0.0005**
	**≤25**	397 (18.5)	1754				206 (9.6)	1945			
2018	**>25**	267 (17.5)	1259	0.97	0.81–1.16	0.77	n/a	n/a	n/a	n/a	n/a
	**≤25**	359 (17.9)	1642				n/a	n/a			
2019	**>25**	327 (20.8)	1243	1.16	0.98–1.38	0.08	68 (4.3)	1502	0.48	0.35–0.64	**<0.0001**
	**≤25**	378 (18.5)	1669				178 (8.7)	1869			
2020	**>25**	326 (19.5)	1342	1.08	0.91–1.28	0.4	89 (5.3)	1579	0.71	0.54–0.94	**0.02**
	**≤25**	363 (18.4)	1610				145 (7.3)	1828			
2021	**>25**	332 (20.2)	1312	1.12	0.95–1.33	0.19	80 (4.9)	1564	0.63	0.47–0.84	**0.001**
	**≤25**	355 (18.4)	1573				145 (7.5)	1783			

^a^ Chi-square test, significant values are highlighted by bold text.

### Endoscopic management

**Figure 3.**
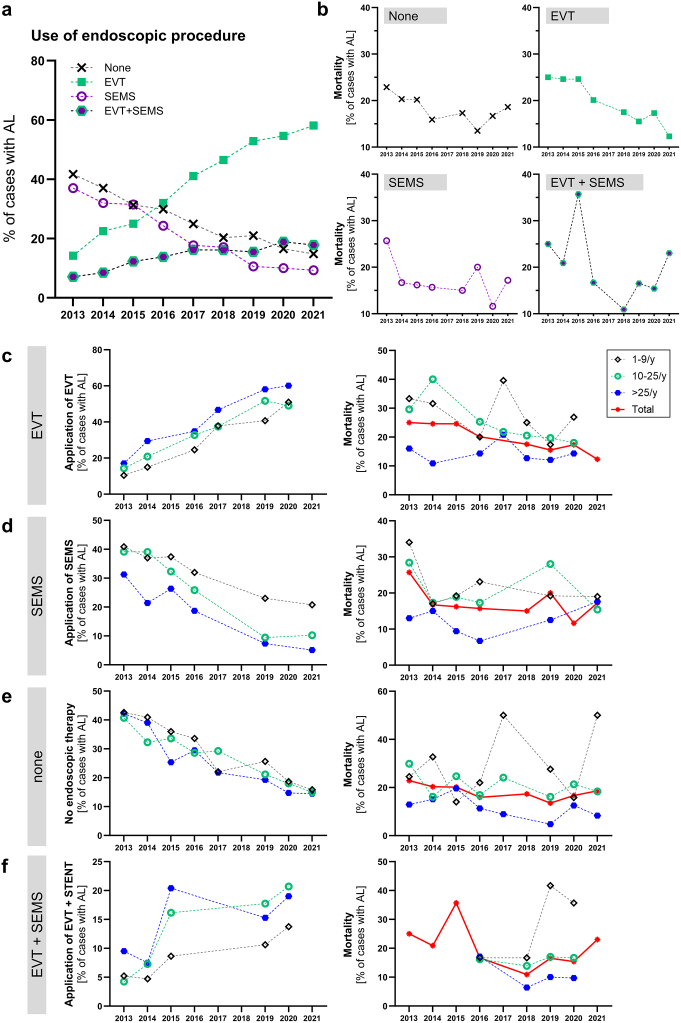
Endoscopic management of anastomotic leakage. (a) Application rates for endoscopic vacuum therapy (EVT, green squares), endoscopic stenting (SEMS, purple circle), a combination or consecutive use of EVT and SEMS (hexagon), and no documented specific endoscopic treatment (diagonal cross) per year in cases with anastomotic leakage (AL). (b) In-house mortality rates for cases with AL and specific endoscopic management. (c) Application rates of EVT in cases with AL according to hospital case volume (low-volume centers (≤9 cases/year, black rhombus), medium-volume centers (10–25 cases/year, green circle), and high-volume centers (>25 cases/year, blue hexagon)). (d) Application rates of SEMS in cases with AL according to hospital case volume. (e) No specific endoscopic therapy coded in cases with AL according to hospital case volume. (f) Application rates of EVT and SEMS in cases with AL according to hospital case volume.

Regarding endoscopic management of AL, cases with AL and EVT, SEMS, a combination of EVT and SEMS, and no coded endoscopic treatment were analyzed. A considerable increase in the use of EVT was seen, and 14.2% of AL cases were treated with EVT in 2013 and 58.1% in 2021. Accordingly, case numbers with SEMS treatment for AL decreased from 37.9% in 2013 to 9.3% in 2021. Cases in which both procedures were performed increased from 7.1% in 2013 to 17.8% in 2021. Cases in which none of these procedures were documented decreased from 41.7% in 2013 to 14.8% in 2021 (Fig. 3a). Mortality rates in cases with EVT showed a steady decrease during the study period with a mortality rate of 25.0% in 2013 and 12.3% in 2021 (Fig. 3b). Trends toward lower mortality rates for the other treatment modalities were not obvious.

The use of EVT increased over time for the entire study population, with the highest rates of use in high-volume centers and the lowest rates in low-volume centers (Fig. 3c). Mortality in cases treated with EVT differed according to the center volume. While mortality rates were stable in high-volume centers (16.0% in 2013, 14.3% in 2020), mortality decreased from 29.6% in 2013 to 18.0% in 2020 in medium-volume centers for cases with EVT (Fig. 3c).

Inversely to the use of EVT, application rates of SEMS were highest in low-volume centers and lowest in high-volume centers (Fig. 3d). In-house mortality rates for cases with AL and the application of SEMS were lowest in high-volume centers (Fig. 3d).

As described above, the number of cases in which the use of neither EVT nor SEMS was documented decreased over the study period, with no obvious difference between centre volumes (Fig. 3e).

The combination or consecutive application of EVT and SEMS was most commonly applied in medium- and high-volume centers, while mortality again was lowest in high-volume centers (Fig. 3f).

### Hospital length of stay and socioeconomic costs

As expected, the hospital length of stay was generally longer in cases with AL compared to cases without AL. In cases without AL, a trend toward shorter hospital stays was seen for all center sizes (mean length of hospital stay: low-volume centers: 25.9 days in 2013, 26.5 days in 2016, 22.9 days in 2021; medium-volume centers: 27.5 days in 2013, 24.4 days and 22.0 days in 2021; high-volume centers: 25.5 days in 2013, 23.5 days in 2016, and 19.8 days in 2021; Fig. S2a, http://links.lww.com/JS9/D811). For patients with AL, only in high-volume centers, a discernible decrease in the length of hospital stay was observed with a mean length of hospital stay of 51.1 days in 2013 and 43.6 days in 2021 (Fig. S2a, http://links.lww.com/JS9/D811). The mean hospital reimbursement from 2013 to 2021 was €55 738 per case for cases with AL, while the mean hospital reimbursement for cases without AL was €26 267 per case (Fig. S2b, http://links.lww.com/JS9/D811).

We calculated the hypothetical savings if no AL would occur after esophagectomy (Table S4, http://links.lww.com/JS9/D811). By preventing AL, the hypothetical annual savings would have been between 10 956 860 and 25 081 476 Euro (mean 18 116 720 Euro) (Table S4, http://links.lww.com/JS9/D811).

## Discussion

We present here the first secondary data analysis of nationwide hospital reimbursement data for esophagectomy in Germany with regard to AL. We focused on in-hospital mortality associated with AL and recent developments in endoscopic treatment of AL related to annual hospital case volume. The unique feature of this study is that there is no selection bias, as all hospital cases in Germany were included, not only cases from certified centers or academic hospitals. Briefly summarized, the data show that AL rates are not dependent on hospital volume for esophagectomy when comparing hospitals with an annual volume of ≤ 25 cases to hospitals with >25 cases per year (OR 1.06, 95% CI 0.99–1.12, *P* = 0.07); however, in-hospital mortality is significantly lower in high-volume centers with >25 cases per year (OR 0.60, 95% CI 0.54–0.66, *P* < 0.0001; Table 2). However, it is noted that reported AL rates are still disproportionately high in the study population with a mean reported AL of 19.2% in 2021. Still, overall mortality rates decreased during the study period (8.6% in 2013 to 6.3% in 2021); however, in cases with AL, the mean in-house mortality was about three times higher than in cases without AL (18.9% vs. 4.7%). The association of yearly hospital case volume and in-house mortality rates has been reported previously by Nimptsch and colleagues[17] who also used a secondary data analysis of the G-DRG database. Based on their results, the minimum required yearly volume of complex esophageal surgeries has been increased by the GBA from 10 to 26 recently. However, in their analysis, they were only able to evaluate AL as a specific postoperative complication indirectly by analyzing the number of endoscopic interventions as the ICD code for anastomotic leakage was only introduced to the German DRG system in 2013, highlighting the added value of our study to the topic.

Failure to rescue, defined as the mortality of patients with major postoperative complications such as AL, has emerged as an indicator of the quality of postoperative care. It is increasingly used to describe relationships between hospital volume and postoperative outcomes[27]. The mean in-house mortality rate for cases with AL (failure-to-rescue rate) in the present study was 18.9% with an inverse correlation regarding hospital volume. Abdelsattar *et al* reported a similar failure-to-rescue rate of 18.5% based on a major complication rate after esophagectomy of 26.6% from a retrospective analysis of 26 820 patients from the American Agency for Healthcare Research and Quality Nationwide Readmission Database; however, they did not report on specific failure-to-rescue rates with regard to AL[28]. Harris *et al* also reported a failure-to-rescue rate after esophagectomy of 11% in esophagectomy-targeted vs. 17% in standard hospitals based on the American College of Surgeons (ACS) National Surgical Quality Improvement Program (NSQIP) esophagectomy-targeted registry demonstrating a lower failure-to-rescue rate in hospitals participating in the NSQIP esophagectomy-targeted registry[29]. In a multicenter, retrospective cohort study including 1509 patients led by the TENTACLE–Esophagus study group, the failure-to-rescue rate after AL was 11.7%, with a lower failure-to-rescue rate in middle- and high-volume centers[30]. Endoscopic treatment is the preferred therapeutic modality to treat cases with AL when revision surgery can be avoided and when aiming to preserve the anastomosis. Since 2012, EVT has been increasingly applied for the management of AL after esophagectomy in Germany and has even been applied as a preventative method to reduce the risk of AL in high-risk patients^[31,32]^. In our data analysis, we found that the use of EVT increased from 14.2% of cases with AL in 2013 to 58.1% of cases in 2021. Correspondingly, the percentage of cases with AL treated with SEMS or with neither EVT nor SEMS decreased (Fig. 3A). One could speculate that the addition of EVT to the potential rescue options and its increasing use for cases with AL may be associated with a decrease in all-cause and AL-related in-hospital mortality, but causal conclusions cannot be drawn from the present data. Due to the retrospective nature of the dataset analyzed in the study and the missing information on a variety of other possible causes of decreased mortality, such as strategies to detect AL, use of conservative treatment options (antibiotics, parenteral nutrition, etc.), and severity of leakage, reliable conclusions about a true causal relationship between the use of EVT and decreased mortality cannot be made based on the DRG statistics data.

Two meta-analyses have analyzed several retrospective studies comparing EVT and SEMS with regard to the treatment success for AL after esophagectomy^[9,10]^. Only the first showed an advantage of EVT over SEMS regarding mortality rates. Regarding these data, it would be highly speculative (as mentioned in the original manuscript) to draw causal conclusions about the association between the use of EVT and an overall reduction of in-hospital mortality from the data presented in the present study. This only underlines the need to conduct high-quality prospective, multi-center randomized controlled trials on the effect of endoscopic rescue procedures and failure to rescue after esophagectomy. Interestingly, however, the increased application of EVT did not lead to an increase in the length of hospital stay, which actually decreased during the study period Supplemental Online Material (Figure S2a, htt p://links.lww.com/JS9/D811. For robot-assisted esophagectomies, AL rates were significantly higher from 2014 to 2016 and then decreased in 2017 to levels similar to those for non-robot-assisted esophagectomies (Table S3, http://links.lww.com/JS9/D811, Figure S1, http://links.lww.com/JS9/D811). However, at the end of the study period in 2021, AL rates were still higher for robotic-assisted esophagectomies (22.9% vs. 18.3%; Table S3, http://links.lww.com/JS9/D811). The high AL rates during the introduction of robotic surgery can most likely be attributed to the learning curve of the new surgical procedure, but the robotic approach did not lead to an overall decrease in AL rates. To our knowledge, real-world data comparing AL rates from robotic-assisted vs. conventional (open, minimally-invasive) esophagectomies have not been reported yet. Meta-analysis comparing robotic-assisted with minimally-invasive approaches did not show differences in AL rates^[33,34]^. This highlights the importance of reporting real-world data using nationwide statistical data. In addition, the data suggest that there may be value in considering national quality control measures, particularly when introducing novel surgical techniques such as robotic surgery for procedures with inherent complexity, such as esophageal surgery. This could help ensure that optimal patient care is consistently provided.

### Limitations

Despite the advantage of excluding selection bias for reported cases, there are several limitations to the study, as discussed in the following. Although this data analysis includes all inpatient cases treated in a German acute care hospital and thus represents a large study population, it has some limitations, as previously reported[35]. A major disadvantage of the type of data analyzed is that the analysis is based on hospital reimbursement data and not on individual longitudinal analysis of individual patient cases. As such, individual patient outcomes such as successful treatment of AL and cancer-specific data cannot be analyzed. In addition, information on the general condition of the patients or the severity of the infection after the leakage, the timing of the rescue treatment and the sequential strategy for the rescue cannot be derived from the data set. As previously reported, this type of data analysis has an inherent risk for under- or overreporting of certain ICD codes and different coding practices in individual hospitals. However, the risk of over- and underreporting is reduced through external validation through the Health Insurance Medical Services (to avoid overreporting to avoid additional costs for health insurance providers) and internal validation by hospital documentation staff (to avoid underreporting to avoid financial losses for individual hospitals). As there is no specific code for revision surgery during the initial hospital stay, rates for revision surgery after esophagectomy and associated mortality could not be analyzed in this study.

## Conclusions

To conclude, the present data reveal that AL rates remain very high with no improvement over a 9-year period. In-house mortality is slowly decreasing. Despite the known risk reduction regarding in-house mortality by centralization of complex surgeries such as esophagectomies, more than half of the patients are still treated in low- and medium-volume centers.

Despite the challenges of centralizing certain surgical procedures, it would be an effective option to reduce overall mortality after esophagectomy in Germany, and our data underscore the importance of pursuing centralization efforts led by central health care administrators in Germany and other countries with comparable health care systems.

Given the alarmingly high rates of AL, future research must focus on further improving the technical surgical aspects of anastomosis formation but also invest in understanding the biology of anastomotic healing itself to prevent postoperative pathological processes leading to AL. In addition, efforts must be made to optimize treatment strategies for severe complications such as AL to improve patient outcomes even in settings where centralization is not feasible for reasons such as availability and access to care for patients requiring esophagectomy.”

## Data sharing statement

The SAS code that was used to request data from the German Federal Statistical Office (DESTATIS) can be requested through the corresponding author.

## Presentation

Preliminary data was presented at “Deutscher Chirurgie Kongress” (DCK) 2024, Leipzig.
